# Persistence of COVID-19 Symptoms after Recovery in Mexican Population

**DOI:** 10.3390/ijerph17249367

**Published:** 2020-12-14

**Authors:** Carlos E. Galván-Tejada, Cintya Fabiola Herrera-García, Susana Godina-González, Karen E. Villagrana-Bañuelos, Juan Daniel De Luna Amaro, Karla Herrera-García, Carolina Rodríguez-Quiñones, Laura A. Zanella-Calzada, Julio Ramírez-Barranco, Jocelyn L. Ruiz de Avila, Fuensanta Reyes-Escobedo, José M. Celaya-Padilla, Jorge I. Galván-Tejada, Hamurabi Gamboa-Rosales, Mónica Martínez-Acuña, Alberto Cervantes-Villagrana, Bruno Rivas-Santiago, Irma E. Gonzalez-Curiel

**Affiliations:** 1 Unidad Académica de Ingeniería Eléctrica, Universidad Autónoma de Zacatecas, Jardín Juarez 147, Centro, Zacatecas 98000, Mexico; ericgalvan@uaz.edu.mx (C.E.G.-T.); kvillagrana@uaz.edu.mx (K.E.V.-B.); jose.celaya@uaz.edu.mx (J.M.C.-P.); gatejo@uaz.edu.mx (J.I.G.-T.); hamurabigr@uaz.edu.mx (H.G.-R.); 2 Unidad Académica de Ciencias Químicas, Universidad Autónoma de Zacatecas, Jardín Juarez 147, Centro, Zacatecas 98000, Mexico; cintyafhg77@hotmail.es (C.F.H.-G.); sgodina@uaz.edu.mx (S.G.-G.); kihg7@hotmail.com (K.H.-G.); karolina.roqui@gmail.com (C.R.-Q); fuenreyes@uaz.edu.mx (F.R.-E.); monicaimeldamtza@uaz.edu.mx (M.M.-A.); dr.albertocervantes@uaz.edu.mx (A.C.-V.); 3 Hospital General de Jerez, Zacatecas 99390, Mexico; danieldelunaa@gmail.com; 4 LORIA (INRIA, CNRS, Université de Lorraine), Campus Scientifique BP 239, 54506 Nancy, France; laura.zanella-calzada@univ-lorraine.fr; 5 Epidemiología y Medicina Preventiva, ISSSTE, Gpe. Zacatecas 98613, Mexico; julio.ramirezb@issste.gob.mx; 6 Facultad de Medicina, Centro de Investigación en Ciencias de la Salud y Biomedicina (CICSaB), Universidad Autónoma de San Luis Potosí, San Luis Potosí 78300, Mexico; sakuliz_12@hotmail.com; 7 Unidad de Investigación Biomédica de Zacatecas, IMSS, Zacatecas 98000, Mexico; rondo_vm@yahoo.com

**Keywords:** COVID-19, SARS-CoV-2, post-COVID syndrome, recovered from COVID, persistent symptoms

## Abstract

The severe acute respiratory syndrome coronavirus 2 (SARS-CoV-2) is responsible for the coronavirus disease (COVID-19), a highly contagious infectious disease that has caused many deaths worldwide. Despite global efforts, it continues to cause great losses, and leaving multiple unknowns that we must resolve in order to face the pandemic more effectively. One of the questions that has arisen recently is what happens, after recovering from COVID-19. For this reason, the objective of this study is to identify the risk of presenting persistent symptoms in recovered from COVID-19. This case-control study was conducted in one state of Mexico. Initially the data were obtained from the participants, through a questionnaire about symptoms that they had at the moment of the interview. Initially were captured the collected data, to make a dataset. After the pre-processed using the R project tool to eliminate outliers or missing data. Obtained finally a total of 219 participants, 141 recovered and 78 controls. It was used confidence level of 90% and a margin of error of 7%. From results it was obtained that all symptoms have an associated risk in those recovered. The relative risk of the selected symptoms in the recovered patients goes from 3 to 22 times, being infinite for the case of dyspnea, due to the fact that there is no control that presents this symptom at the moment of the interview, followed by the nausea and the anosmia with a RR of 8.5. Therefore, public health strategies must be rethought, to treat or rehabilitate, avoiding chronic problems in patients recovered from COVID-19.

## 1. Introduction

The health emergency caused by a novel virus, designated as severe acute respiratory syndrome coronavirus 2 (SARS-CoV-2), which is responsible for coronavirus disease (COVID-19), highly contagious infectious disease that has caused more than 38 million cases and more than a million deaths in approximately 150 countries [1]. Since February 2020 from to date, in Mexico, have been reported more than 800,000 confirmed cases and more than 84,000 deaths by this disease [2], numbers that continue increasing daily.

This disease is generally characterized by a respiratory condition, also the literature [3,4,5] mentions the existence of a wide range of manifestations, ranging from people asymptomatic, since to serious clinical pictures that require intensive care and others who unfortunately die from this disease.

According to the World Health Organization (WHO) [6], to date, for suspected cases both epidemiological and clinical criteria are met. The clinical criteria include; acute onset of fever plus cough. Or acute onset of three or more signs or symptoms such as fever, cough, asthenia (fatigue), cephalgia (headache), myalgia (muscle pain), odynophagia (sore throat), coryza (rhinorrhea or nasal discharge), dyspnea (difficulty breathing), anorexia (loss of appetite)/nausea/vomiting, diarrhea, or mental disorders. However, any case that presents severe acute respiratory disease (SARI: acute respiratory infection with history of fever or measured fever ≥38 °C; and cough; starting in the last 10 days; and requiring hospitalization), should be considered a suspect case immediately [6].

There are also probable cases, which are based on the presence of clinical criteria, but with links to already confirmed or probable cases, Another way to consider it probable is with other symptoms such as dysgeusia (loss of taste) and anosmia (loss of smell), or an unexplained death (patient has presented respiratory distress before death), among other criteria [6].

The confirmed cases of COVID-19 will be the people who present a laboratory confirmation of COVID-19 infection [6]. In the case of Mexico, a patient is considered to have recovered, if fourteen days have elapsed since the onset of symptoms [7].

These definitions may vary over time, because we are faced with a new disease, which is in continuous scientific research [8]. It is still unknown about this disease, especially what happens after a patient is discharged and diagnosed as a recovered case of COVID-19. Reports related to this are briefly discussed in the next Section 1.1.

### 1.1. Related Work

Various investigations have been developed, which have been focused on various aspects of this pandemic. Some of them regarding the part epidemiological [9,10], others about the clinical issue [11], organ and system complications different to respiratory how cardiovascular system and myocardial damage, nervous and digestive affectation [12,13,14,15,16,17,18,19,20], symptomatology characteristic [9,21], risk groups [11], and recently, on persistence of symptoms in patients apparently recovered from COVID-19.

In the literature [22], it was expected that SARS-CoV-2 would induce a monophasic disease with at least transient immunity, however, in the face of suspicion of recurrence, or reactivation of COVID-19, after discharge, questions have arisen due to reports of persistence of symptoms, as shown in studies from China, the United States of America, France, Ireland, among others [22,23,24,25,26,27,28].

In Italy, Carfì et al. [25], worked about a study on the persistence of symptoms in recovered patients from COVID-19. Finding that 87.4% reported persistence of at least 1 symptom and 55% of patients had 3 or more symptoms. Highlighting asthenia and dyspnea as the main symptoms. However, one of the shortcomings of this study is that it did not present control cases.

In this same sense, the work proposed by Lamprecht [26], mentions that it is possible to speak of a post-COVID syndrome, sharing with other works [25], the persistence of symptoms of fatigue and dyspnea; also includes mental health problems and long-term quality of life problems. On the other hand, this author questions the term, since it is too early to diagnose a syndrome post-COVID, since it would be necessary for this, at least a period of 6 months with persistent symptoms, therefore the term post-infection asthenia could currently be used. However, in the literature, it seems to be used interchangeably, for example neurological and immunologic manifestations have also been described as part of the post-COVID syndrome [29]. Studies in Greece have also reported recovered cases of COVID-19, with persistent symptoms, anosmia and dysgeusia being common [28].

In the United Kingdom, a study was carried out in patients hospitalized for COVID-19, both those who required a stay in the intensive care unit (ICU), and those hospitalized in the ward, finding asthenia, with a frequency of 72% in the ICU group and 60% in the ward group, dyspnea (65% in patients in the ICU group and 42% in the ward group), in addition they found psychological alterations in both groups [28].

After having carried out two tests, which must be negative, as the literature suggests [30], for the diagnosis of a recovered patient, it is essential to know why some studies [31,32] have reported that after having obtained these two negative tests, patients present symptoms and present in some cases positivity of new tests. Because this phenomenon has been described in different countries [24,33,34], it is discussed that cannot be attributed only to false negatives due to the coincides with others studies, so it could be a reactivation or reinfection [30].

It is clear that, there is still much to discover about COVID-19, because it continues to be active in all the world. As a consequence is necessary to continue investigating to understand and find solutions that mitigate, complications regarding the health of patients recovered, such short, medium and long term. So this work focused in the persistence of symptoms in recovered patients of COVID-19, to know if they are present, and which of them are those that predominate.

This paper is organized as follows. In Section 2 a detailed description of the data set used for this study is presented, as well as the methods applied to analyze the population and symptoms. In the Section 3 the experiments and results of the methods used to determine the risk of symptom recurrence in patients recovered from SARS-CoV2 are shown. The results obtained are discussed dynamically in the Section 4, as well as conclusions in terms of risk.

## 2. Materials and Methods

This section describes in detail the design of the study, the methodology used, as well as the participants involved in this work.

**Figure 1:**
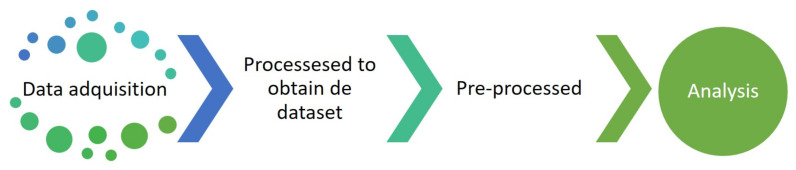
Flowchart of the methodology followed.

In Figure 1 the methodology followed is shown. Initially the data are obtained from the participants, processed to obtain the data set, and then pre-processed using the R project tool to eliminate outliers or missing data and finally perform the symptom analysis.

### 2.1. Study Design

This case-control study was conducted in the state of Zacatecas Mexico, which has a population of approximately 1.5 million people, with a population of 5432 Covid-19 recoveries at the time of recruitment. Data was collected from 25 July to 20 September 2020.

### 2.2. Participants

The participants belong to only 3 particular communities in Zacatecas, being the city of Zacatecas, the city of Guadalupe and the conurbated area between them, representing a total population of approximately 400,000 people.

The sample size of subjects who participated in the questionnaire and agreed to be part of the study constituted a total of 270, of which 99 are controls (people who do not meet both clinical and laboratory criteria to diagnose SARS-CoV2) and 171 are recovered subjects (people who had a laboratory-confirmed diagnosis of SARS-CoV-2, and in whom at least fourteen days have passed since the appearance of symptoms.)

Inclusion criteria: residence in a metropolitan area of Zacatecas, Mexico, for the control group; that there was an absence of criteria to diagnose COVID-19 (both clinical and laboratory). In the group of recovered patients; patients whose COVID-19 diagnosis was confirmed by laboratory were included, and at least fourteen days had elapsed since the onset of symptoms. Exclusion criteria: all persons who did not meet the inclusion criteria.

### 2.3. Composition of the Symptom Questionnaire

For the composition of the symptom questionnaire, the sample of recovered subjects and controls were asked if they currently had any of the following symptoms; (1) fever, (2) myalgia, (3) rhinorrhea or coryza, (4) asthenia, (5) cough, (6) cephalgia, (7) red eyes, (8) odynophagia, (9) nausea, vomit or diarrhea, (10) anosmia or dysgeusia, (11) stomach pain or discomfort, (12) dyspnea, (13) chills.

### 2.4. Pre-Processing

For the pre-processing, two main steps are followed. The first step consists on removing all the samples that presented missing values in all columns. Then, the missing values that remained in the data are imputed using a method based on fully conditional specification, where incomplete features are imputed by separate models. For this step, the “Mice” [35] package is used. This second step is specifically applied to the missing values that are present in the feature concerning the edge of the participants. For the features concerning the symptoms, no missing values are present, since, acting as binary features, they refer to “1” when a participant says they suffer a symptom, and “0” otherwise.

After pre-processing, a sample of 219 participants (141 recovered and 78 controls) meeting the criteria is obtained. This number of samples allows assuming a confidence level of 90% and a margin of error of 7%, considering the population of the region described above. For this step, no difference referring to the demographics of the participants was taken into account as exclusion or inclusion criteria.

Based on this, the set of 13 features mentioned in the composition of the symptom questionnaire are taken into account for the subsequent analysis, each one corresponding to the symptoms of the questionnaire, while in the output, 0 is assigned if the values of the features correspond to a control, and, 1 if the values correspond to a recovered subject.

### 2.5. Modeling

In the modeling stage, the data are submitted to a logistic regression (LR), which is used to model a binary outcome (being possible to extend it to a multiple outcome), where the logs odds of the outcome are modeled as a linear combination of one or more input features. The outcome is represented as an indicator variable, where the possible values given are “0” or “1”. LR can be calculated with Equation (1), where $x_{n}$ represent the input features, $\beta_{i}$ represent the coefficients of the model, and *b* the base of the logarithm.

(1) $$log\left(odds\right)=log_{b}\frac{p}{1-p}=\beta_{0}+\beta_{1}x_{1}+\beta_{2}x_{2}+...+\beta_{n}x_{n}$$

### 2.6. Statistical Evaluation

From the model obtained, the relative risk ratio (RR) is calculated for each feature.

All the methodology is developed in R [36], “a free software environment for statistical computing and graphics”.

## 3. Experiments and Results

**Figure 2:**
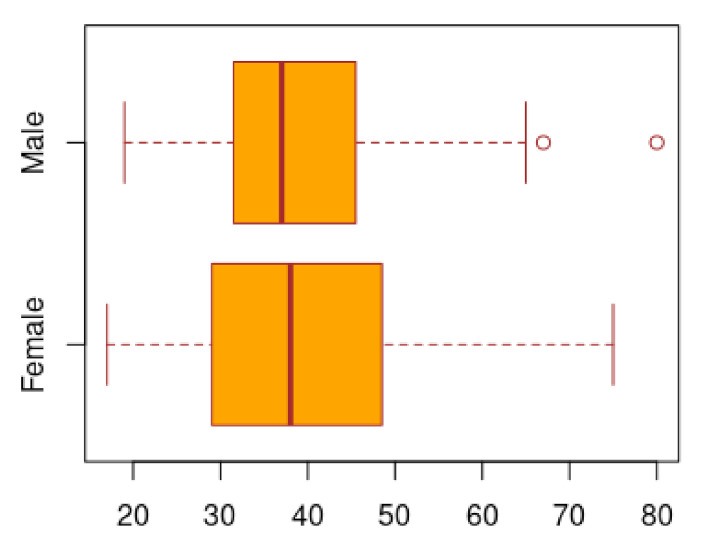
Age distribution in females and male patients.

From total of 219 participants, 141 recovered and 78 controls allows to achieve the proposed sample size to assume a confidence level of 90% and a margin of error of 7%. This sample is comprised by 51% females and 49% males. In Figure 2 shows their age distribution by sex, with a mean of 39.14 for females and 39.01 for males. The symptom persistence reported for subjects with at least one symptom has an average of 31.23 days with a minimum of 1 day and a maximum of 60 days as presented in Table 1. Specific to recovered subjects has an average of 32.63, with a minimum of 2 days and a maximum of 60 days. Additionally, observed recovered time has a minimum of 14 days (as is requested by Mexican federal government [7]), to 176 days post infection, with a mean of 36.07 days.

Regarding the controls, 51 of them were free of COVID-19 associated symptoms at the time of the interview, while 27 had at least one of the most significant symptom, which means that the risk of symptoms in the controls is 0.3461, that is 34.61%, while for the recovered population, 22 have no symptoms and 119 have at least one symptom, that is, a risk of 0.8439 (84.39%) as is shown in Table 2. Meaning that 0.6438 (64.38%) of studied population is recovered.

With the above, the risk ratio associated with having the recovered status is calculated, as shown in the following Table 3.

Therefore, recovered RR is 2.43 CI (1.78, 3.33) of presenting any symptoms. Odds ratio are in the same direction as is presented in Table 4. Thus, odds ratio (OR) are 10.21717 CI (5.325675, 19.60139), both of them (RR and OR) with a *p* value less than 0.001.

Once the RR and the OR are evaluated for the recovered patients, knowing that they have a significant value, an analysis is made to know which is the impact of each one of the symptoms in the recovered patients.

The set of samples is then subjected to a multinomial logistic regression approach, where the full group of features are modeled in relation to the output. The output contains two classes, which correspond to: control (0) or recovered (1).

After modeling the data, a statistical evaluation is applied in order to measure the contribution of each feature in relation to the output. For this purpose, the RR values calculated. In Table 5 are listed the set of symptoms that were modeled, which are arranged in ascending order according to the RR value obtained.

From the above evaluation using a logistic regression modeling approach, it is obtained that all the symptoms contribute giving a positive risk, therefore, symptoms reported in the literature as present or recurrent in patients recovered in different works [22,23,24,25,26,27,28] were selected to be evaluated independently, in terms of absolute as well as relative risk, in order to evaluate and compare persistent symptoms reported internationally. In Table 6 is presented the number of controls and recovered that suffers of one or more symptoms at the moment of the interview.

In Table 7 is shown how individually for each of the symptoms, risk is observed, being for all cases a higher risk in recovered patients.

Finally, the relative risk is greater than 1 for all the selected symptoms, being the dyspnea the only one with a tendency to infinity since there are no controls with this symptom, shown in the Table 8.

Anosmia, as well as nausea or vomiting are the two symptoms that present the greatest risk of recurrence in recovered patients, and in particular, dyspnea is a symptom that is only present in recovered patients.

## 4. Discussion and Conclusions

The results of this study on persistent symptoms in patients recovered from COVID-19 coincide with regional studies from other countries, such as those carried out in Italy, Germany, the United States and others [22,23,24,25,26,27,28]. Referring to the presence of a post-COVID syndrome in which symptoms persist with different frequency after recovering from their initial illness. Several studies have analyzed different symptoms or conditions that are presented in recovered patients, exhibiting multi-organ manifestations like into the gastrointestinal tract, kidney, heart, brain, eyes and lungs, respectively, which demonstrate evidence of these collateral damages within recovered patients. The results follow that recovered patients are at increased risk of persistent symptoms similar to those caused by COVID-19 with a high risk compared to control persons.

Regarding the evident difference of the results obtained from OR and RR, in the literature [37], there is debate regarding the merits of the RR compared to the OR for the analysis of trials and cohort and cross-sectional studies with common results, which in our case the result is attributed to the fact that the persistence of symptoms after COVID-19 infection was common. Since if the result of a study is common, the OR will be farther from 1 than the RR [37].

Given that all symptoms have an associated risk in those recovered, from the selection of symptoms it is possible to observe that the absolute risk of the controls may be due to a number of different factors, with cough (5%) being the symptom of greatest absolute risk within the controls. However, it is worth mentioning that the absolute risk in recovered patients is 3 times higher (15%).

Respect for our results, one of the symptoms more important is the dyspnea, since among the reported symptoms it is the only one that is not present in any of the controls, being important as a recurrent descriptive symptom. This symptom supports, that attention should be paid to prevent or treat complications, one of the most important, pulmonary fibrosis. This alterations is a form of interstitial lung disease, in which lung parenchyma is replaced by scar tissue, making gas exchange difficult.

Since it has been reported in the literature that equal than other coronaviruses are responsible for producing pulmonary fibrosis [38,39,40,41], and that this has also been recognized as a sequel to acute respiratory distress syndrome (ARDS). Also, dyspnea related with COVID-19 can be associated with heart problems [42,43], so its origin should be investigated, and recovered patients should be followed up, since the extent of the complications is still unknown [44].

It has also been described that symptoms such as dyspnea, anosmia, have appeared in survivors of severe acute respiratory syndrome (SARS-CoV) in 2003, where the individuals presented persistent functional disability, after discharge, it stands out that they were young patients, it is even mentioned that cases were registered, that they presented debilitating symptoms after one year of apparent recovery, and the possibility that these sequelae, were neurological, caused by infection or inflammation in the central nervous system, is discussed in other research [45]. Anosmia is an important symptom, because it could be indicate of intranasal inoculation of SARS-CoV-2 into the olfactory neural circuitry causing a neuroinvasion that could result in chronic neurodegenerative disease [46,47,48]. However, it’s necessary to make research in this area to uncover the mechanism of SARS-CoV-2 infection via olfactory bulb and its implications in neurodegenerative diseases the long term [48].

The relative risk of the selected symptoms in the recovered patients goes from 3 to 22 times, being infinite for the case of dyspnea, that as it was mentioned this is due to the fact that there is no control that presents this symptom at the moment of the interview followed by nausea and the anosmia with a RR of 8.5.

Regarding gastrointestinal problems, it has been reported that [49] are predictors of severe cases of COVID-19, which could be related to the degree of replication and viral load, and this influence damage to organs and systems, and manifest in the persistence of residual symptoms. Likewise, clinical cases of multisystemic inflammation syndrome have been reported, both in children and adults, in post-COVID-19 patients [50,51]. Agreeing that it is a much more complex multisystem disorder, and refuted what was initially believed, that COVID-19 was a respiratory disease, but now we know that this is not the case [52,53].

To conclude, there are still vast unknowns that will need to be investigated, some of which concern this investigation, are, why there are persistent symptoms, is to say, if they are an alarm of the body, to show that damage is still occurring, or are consequence of the weakening of the systems, due to the confrontation that was had with SARS-CoV-2 infection. Another question is, how long do residual post-COVID symptoms last and if they permanently affect quality of life.

For which it is necessary to known the short, medium and long-term scope of the possible physical and psychological consequences post-COVID, including in these questions, if the times set for social isolation are sufficient [16,54], also about the possible complications, when patients are discharged, that possibly require specific rehabilitative care. It is also in the interest of public health to design strategies that make it possible to face both the problems of the active pandemic and, at the same time, treat recovered cases, but which present complications, to prevent them from becoming chronic and avoid repercussions on the quality of life.

Undoubtedly, one must continue questioning, to find knowledge that contributes to understanding how this disease develops, what consequences it can produce and find the ideal way to face it, obtaining the minimum side effects.

### Limitations of the Study

Within the limits in this study, are; that the symptoms reported by the subjects were obtained through a questionnaire, so the symptoms are subjective according to the perception of each individual.

The study has a relatively young population average (39 years old), so it is unknown if there are variations in persistent symptoms according to age, however, it supports the fact that being young patients and having an epidemiologically lower propensity to chronic diseases degenerative symptoms, the symptoms presented, have a lower risk of being secondary to these chronic diseases.

In the analysis, the subjects were not stratified according to the recovery time, nor if they were patients with severe, moderate, mild symptoms or asymptomatic, which could have contributed to knowing the frequency and types of symptoms persist according to the subgroups of population.

Finally, the cases were taken as recovered, according to the epidemiological definition of our country [7]. Therefore no laboratory tests were performed to corroborate negativity.

## Figures and Tables

**Table 1:** Reported symptoms persistence in days.

Days Interval	Subjects
1	2
2	3
5	2
7	23
15	26
30	47
60	43

**Table 2:** Participants groups divided by condition of No symptoms and Symptoms at the moment of the interview.

Group	No Symptoms	With Symptoms	Total
**Controls**	51	27	78
**Recovered**	22	119	141
**Total**	73	146	219

**Table 3:** Risk ratio related to symptoms in recovered patients. Risk ratio with 95% C.I.

Group	Estimate	Lower	Upper
**Controls**	1	NA	NA
**Recovered**	2.438	1.782621	3.334713

**Table 4:** Odds ratio related to symptoms in recovered patients. Odds with 95% CI.

Group	Estimate	Lower	Upper
**Controls**	1	NA	NA
**Recovered**	10.217	5.326	19.601

**Table 5:** RR of the full set of symptoms.

	Feature	RR
1	Fever	0.124
2	Myalgia	0.319
3	Rhinorrhea	0.870
4	Asthenia	1.307
5	Cough	1.330
6	Cephalgia	1.925
7	Red Eyes	2.190
8	Odynophagia	2.329
9	Nausea, vomit or diarrhea	2.822
10	Anosmia or dysgeusia	3.592
11	Stomach pain or discomfort	4.189
12	Dyspnea	6.923
13	Chills	9.569

**Table 6:** Symptoms present in the cases and controls on the day of the interview.

	Chills	Dyspnea	New Anosmia or Dysgeusia	Nausea or Vomiting	Cough	Red Eyes
**Controls**	6	0	4	1	11	2
**Recovered**	19	14	34	22	35	8

**Table 7:** Absolute risk of groups (controls and recovered) presented the day of the interview.

	Chills	Dyspnea	New Anosmia or Dysgeusia	Nausea or Vomiting	Cough	Red Eyes
**Controls**	0.0274	0.000	0.018	0.004	0.050	0.009
**Recovered**	0.087	0.064	0.155	0.100	0.160	0.036

**Table 8:** Relative risk of recovered participants.

	Chills	Dyspnea	New Anosmia or Dysgeusia	Nausea or Vomiting	Cough	Red Eyes
**Recovered**	3.167	Inf	8.500	22	3.182	4
